# Incidental vertebral fracture prediction using neuronal network-based automatic spine segmentation and volumetric bone mineral density extraction from routine clinical CT scans

**DOI:** 10.3389/fendo.2023.1207949

**Published:** 2023-07-17

**Authors:** Jannis Bodden, Michael Dieckmeyer, Nico Sollmann, Egon Burian, Sebastian Rühling, Maximilian T. Löffler, Anjany Sekuboyina, Malek El Husseini, Claus Zimmer, Jan S. Kirschke, Thomas Baum

**Affiliations:** ^1^ Department of Diagnostic and Interventional Neuroradiology, School of Medicine, Klinikum rechts der Isar, Technical University of Munich, Munich, Germany; ^2^ Department of Diagnostic and Interventional Neuroradiology, University of Bern, Bern, Switzerland; ^3^ TUM-Neuroimaging Center, Klinikum rechts der Isar, Technical University of Munich, Munich, Germany; ^4^ Department of Diagnostic and Interventional Radiology, University Hospital Ulm, Ulm, Germany; ^5^ Department of Diagnostic and Interventional Radiology, School of Medicine, Klinikum rechts der Isar, Technical University of Munich, Munich, Germany; ^6^ Department of Diagnostic and Interventional Radiology, University Medical Center Freiburg, Freiburg im Breisgau, Germany; ^7^ Department of Informatics, Technical University of Munich, Munich, Germany; ^8^ Munich School of BioEngineering, Technical University of Munich, Munich, Germany

**Keywords:** osteoporosis, osteoporotic fractures, bone density, tomography, x-ray computed, artificial intelligence

## Abstract

**Objectives:**

To investigate vertebral osteoporotic fracture (VF) prediction by automatically extracted trabecular volumetric bone mineral density (vBMD) from routine CT, and to compare the model with fracture prevalence-based prediction models.

**Methods:**

This single-center retrospective study included patients who underwent two thoraco-abdominal CT scans during clinical routine with an average inter-scan interval of 21.7 ± 13.1 months (range 5–52 months). Automatic spine segmentation and vBMD extraction was performed by a convolutional neural network framework (anduin.bonescreen.de). Mean vBMD was calculated for levels T5-8, T9-12, and L1-5. VFs were identified by an expert in spine imaging. Odds ratios (ORs) for prevalent and incident VFs were calculated for vBMD (per standard deviation decrease) at each level, for baseline VF prevalence (yes/no), and for baseline VF count (n) using logistic regression models, adjusted for age and sex. Models were compared using Akaike’s and Bayesian information criteria (AIC & BIC).

**Results:**

420 patients (mean age, 63 years ± 9, 276 males) were included in this study. 40 (25 female) had prevalent and 24 (13 female) had incident VFs. Individuals with lower vBMD at any spine level had higher odds for VFs (L1-5, prevalent VF: OR,95%-CI,p: 2.2, 1.4–3.5,p=0.001; incident VF: 3.5, 1.8–6.9,p<0.001). In contrast, VF status (2.15, 0.72–6.43,p=0.170) and count (1.38, 0.89–2.12,p=0.147) performed worse in incident VF prediction. Information criteria revealed best fit for vBMD-based models (AIC vBMD=165.2; VF status=181.0; count=180.7).

**Conclusions:**

VF prediction based on automatically extracted vBMD from routine clinical MDCT outperforms prediction models based on VF status and count. These findings underline the importance of opportunistic quantitative osteoporosis screening in clinical routine MDCT data.

## 1 Introduction

Osteoporosis is a systemic disease that primarily affects bone and is characterized by a quantitative and qualitative decrease in bone substance (1). As a result of impaired bone stability, patients with osteoporosis frequently suffer vertebral (fragility) fractures (VFs), which are associated with a drastic reduction in quality of life and life expectancy (2). Approximately 50% of women and 20% of men experience at least one disease-related fracture during their lifetime, and individuals with a single VF have a 12.6-fold increased risk of further fractures in the future (3–10). Therefore, the history of fractures is queried with the FRAX® questionnaire (http://www.shef.ac.uk/FRAX), an instrument specifically designed to calculate the risk for future VFs (11). However, because clinically silent VFs frequently stay unrecognized, the risk of VFs in those individuals may be drastically underestimated (12). This emphasizes the paramount importance of early-stage diagnosis of osteoporosis and identification of individuals at risk for VFs.

The reference standard for the diagnosis of osteoporosis is given by measurements of bone mineral density by dual X-ray absorptiometry, but the technique’s lower costs and broader availability compared with quantitative computed tomography (CT) are bought by poorer discrimination capabilities between individuals with and without VFs (13–17). In addition, both dual X-ray absorptiometry and dedicated quantitative CT for the purpose of diagnosing osteoporosis require exposure to ionizing radiation and involve additional organizational effort (18–20).

Opportunistic bone densitometry uses imaging data acquired as part of routine clinical practice to determine bone mineral density and is becoming increasingly important as diagnostic CT scans proliferate. While prediction of incident VFs appears feasible in routine CT, region-of-interest segmentation, correction for intravenously administered contrast media, and conversion of Hounsfield Units (HU) to bone mineral density have, until recently, required significant manual effort (21–25). Newly developed deep learning-based tools enable automatic extraction of volumetric bone mineral density (vBMD) from routine clinical CT with specific correction for intravenous contrast phase and specifics of the used CT scanner and protocol (14, 22–26).

However, the performance of models based on automatically extracted opportunistic vBMD measurements to predict VFs, in contrast to established models that account for fracture status and number, is still unclear. Therefore, the aims of this study were to (i) determine cross-sectional associations between opportunistic vBMD and prevalent VFs, (ii) investigate whether vBMD, baseline VF status, and VF count are associated with imminent VFs, and (iii) compare the newly developed vBMD-based fracture prediction models with models based on baseline VF status and number.

## 2 Materials and methods

### 2.1 Study cohort

**Figure 1:**
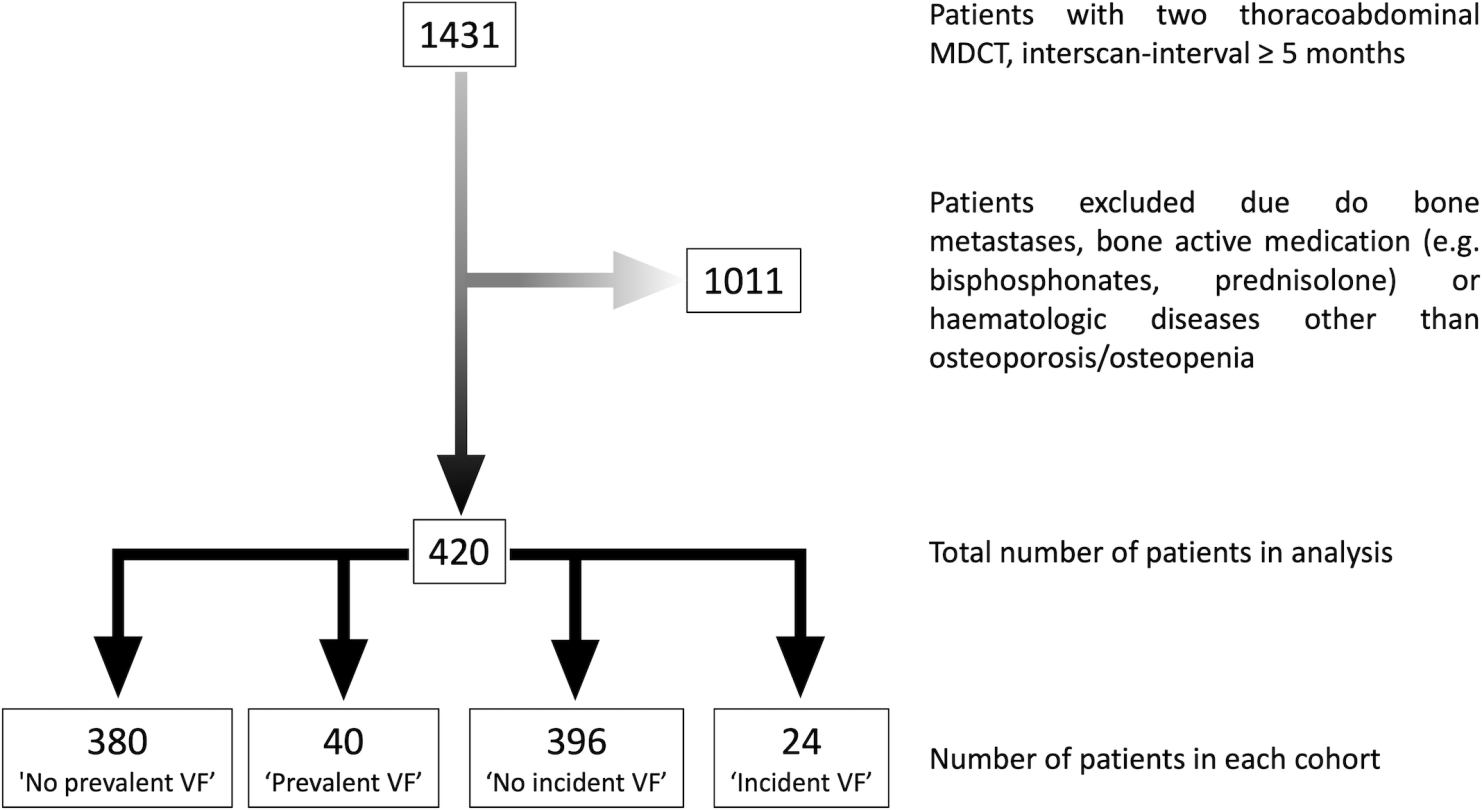
Flow chart depicting the process of patient in- and exclusion.

The institutional ethics committee approved this study and, because of its retrospective nature, waived the requirement for written informed consent. Individuals eligible for participation were retrospectively identified via our institution’s picture archiving and communication system. All individuals had received two thoraco-abdominal multi-detector CT scans at a single site for the purpose of oncologic staging during the six-year inclusion period (**Figure 1**). For each subject, the first scan was considered “baseline” and the second scan was considered “follow-up”. The time interval between both scans had to be at least 5 months.

All scans were manually reviewed for complete coverage of the thoraco-lumbar spine, and subjects with bone metastases, other focal bone lesions of the spine, foreign material implants in the spine with adjacent beam hardening artifacts, or severe motion artifacts were excluded. In addition, all subjects taking bone-active drugs (e. g. bisphosphonates, corticosteroids) or with an established bone disease other than osteoporosis or osteopenia prior to their respective baseline scan were identified and excluded by medical record review.

### 2.2 CT acquisition parameters

All scans were acquired using our institute’s multi-detector CT scanner (Somatom Sensation Cardiac 64; Siemens Healthineers) with 64 detector rows. Tube voltage was set to 120 kVp and the average tube load was 200 mAs. As all scans were performed for oncologic staging, barium-based oral contrast medium was taken approximately 1 h prior to the exam by each individual (Barilux Scan; Sanochemia Diagnostics). For the same reason, all scans were acquired in early venous contrast-phase, performed 70 s after intravenous administration of an iodine-based contrast agent (Fresenius Pilot C; Fresenius Kabi). Intravenous contrast was dose-adjusted for body-weight (≤ 80 kg: 80 ml; 80 kg - 100 kg: 90 ml; > 100 kg: 100 ml) and applied at a flow-rate of 3.0 ml/s. Sagittal views of the spine were reformatted for each scan (slice thickness of 3 mm) using a standard bone kernel.

### 2.3 Automated vertebral body segmentations and BMD measurements

**Figure 2:**
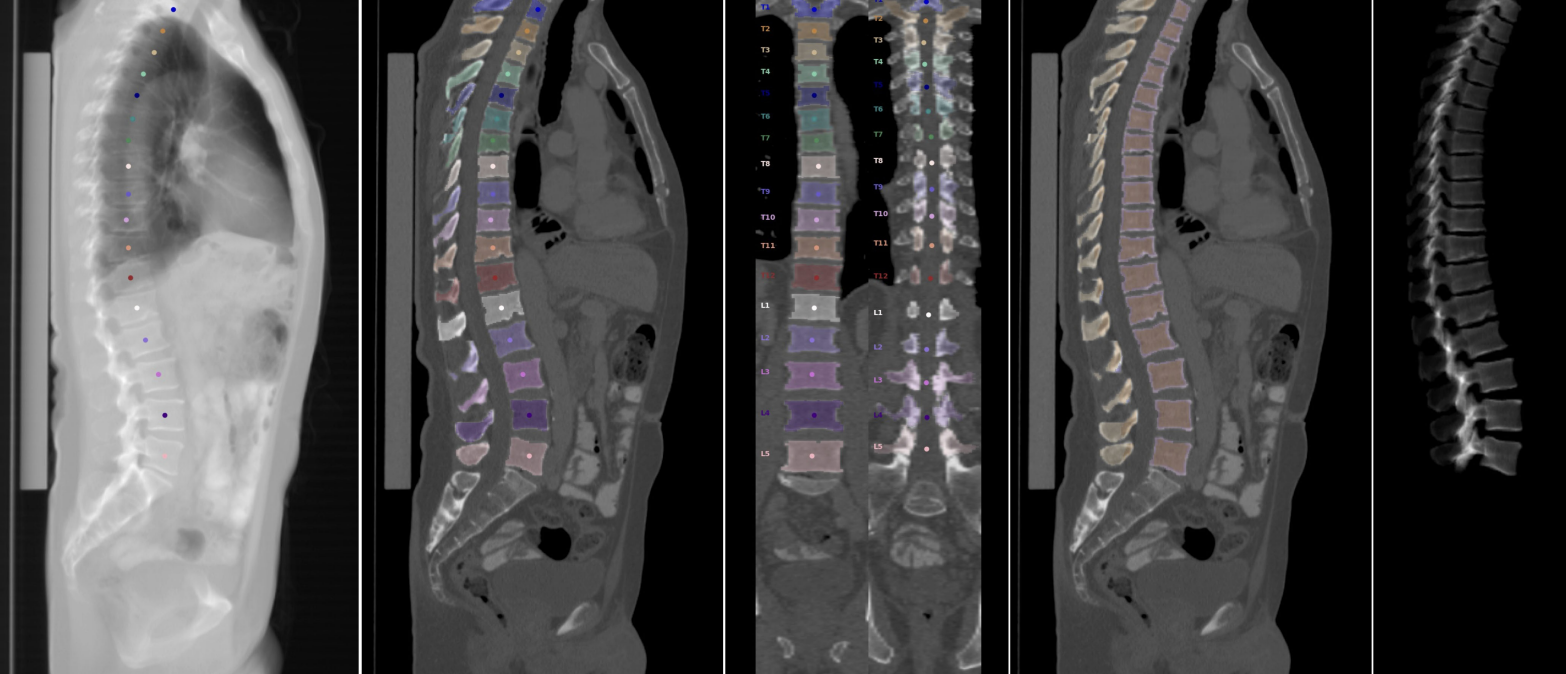
Steps of automated vertebral body segmentation, as performed by the convolutional neural network framework. From left to right: Automated vertebral body detection and labeling. Segmentation of vertebral components, including posterior elements (sagittal and coronal view). Separation of cortical and trabecular bone. Three-dimensional reconstruction of segmented vertebra.

In each scan, the vertebrae of the thoraco-lumbar spine (T1-L5) were automatically labeled and segmented using a convolutional neural network (CNN)-based framework (https://anduin.bonescreen.de), trained on publicly available data (14, 24–29). The tool is based on a deep learning algorithm and creates individual masks for the cortical and trabecular vertebral body compartments as well as laminar regions, facet joints, and processes (**Figure 2**). For the purpose of quality assurance, a neuroradiologist specialized in spine imaging manually reviewed all automated segmentations and corrected them, as necessary (27). Simultaneously, the neuroradiologist further identified and excluded single vertebrae with Modic-type sclerotic endplate changes and intraosseous venous malformations according to the ACR guidelines for quantitative CT (https://www.acr.org/-/media/ACR/Files/Practice-Parameters/qct.pdf).

Based on the aforementioned segmentation masks, trabecular density of each vertebral body was measured in HU. Following asynchronous calibration using a commercially available phantom (QRM QSA-717 Phantom; Quality Assurance in Radiology and Medicine GmbH), HU-to-vBMD conversion was performed using a linear equation model (slope = 0.63). Automated correction for the contrast phase was achieved by implementation of a two-dimensional DenseNet model (26).

### 2.4 VF assessment and cohort definitions

Each subject’s baseline and follow-up scans were manually reviewed for VFs by two readers in consensus (N.S. and M.D., with 9 and 4 years of experience, respectively), adopting a method previously published by Bauer et al. (30). Specifically, VFs were defined as vertebral compression fractures falling into the subcategories A1-4 according to the AO/OTA classification system, and the fracture count was noted for baseline scans (31).

Fractures were classified as ‘prevalent’ if they were present at the baseline scan and ‘incident’ if they were not present at the baseline scan, but at the follow-up scan, i. e., occurred between baseline and follow-up scans. Four cohorts were defined, based on fracture status: ‘no prevalent VF’, ‘prevalent VF’, ‘no incident VF’, and ‘incident VF’. Baseline fracture status did not affect inclusion in the fracture incidence cohorts.

### 2.5 Statistical analyses

All statistical tests were performed using STATA v13.1 software (StataCorp LLC) with a two-sided level of significance of p < 0.05. Mean trabecular vBMD was calculated for three compartments (T5-8, T9-12, and L1-5). Age and sex are both well-known predictors of VFs and osteoporosis, therefore we investigated cohort differences (no prevalent VF’ vs. ‘prevalent VF’ and ‘no incident VF’ vs. ‘incident VF’) using t-tests for age and chi-squared as well as Fisher’s exact tests for sex. If cohort differences were statistically significant (p < 0.05), we included the variables as predictors in our multivariate analyses to address confounding. Furthermore, cohort differences in vBMD at each level were investigated using t-tests.

First, multivariate logistic regression models were used to determine the association between vBMD at each of the three vertebral levels (T5-8, T9-12, and L1-5) and prevalent VFs. Further, the association between the VF count at baseline and vBMD (T5-8, T9-12, and L1-5) was investigated using multivariate linear regression models. In the second part of the analysis, associations between the predictors vBMD (T5-8, T9-12, and L1-5), baseline VF status (yes/no), and baseline VF count and the outcome of incident VFs were also determined using multivariate logistic regression models. Prevalent VF status was stepwise added to all vBMD-based models in the secondary analysis to investigate any confounding.

To simplify model comparisons, all models were also calculated per standard deviation (SD) decrease in vBMD using odds ratio (OR)_SD_, coefficient (Coef)_SD_, and 95%-confidence intervals (CI_SD_). Finally, in order to determine and compare the model fit, Akaike’s and Schwarz’s Bayesian information criteria (AIC & BIC) were calculated for models predicting incident fractures. AIC & BIC are both model performance measures, that aid in model selection, as they reflect how well the individual model represents the data. Lower AIC and BIC values indicate a better fit (32).

## 3 Results

### 3.1 Cohort characteristics

In summary, we identified 1431 individuals who underwent two thoraco-abdominal multi-detector CT scans at the defined scanner for the purpose of cancer staging in the six-year time period. Of those, 1011 were excluded due to bone metastases, use of bone-active medication, and/or hematological diseases other than osteoporosis, diagnosed prior to the baseline scan. Lastly, 420 individuals were eligible for inclusion (276 male, 65.7%). The average age at baseline was 62.9 ± 9.3 years (range 39 to 88 years) and the average inter-scan interval between baseline and follow-up scans was 21.7 ± 13.1 months (range 5 to 52 months). Comprehensive information on demographics of each cohort and fracture distribution is presented in **Table 1**.

**Table 1:** Cohort characteristics.

Variable		unit	no prev. VF	prev. VF	p*	no inc. VF	inc. VF	p*
Age (years)		mean ± SD range	62.5 ± 9.2 39 – 88	67.3 ± 9.8 44 – 82	0.002	62.7 ± 9.3 39 – 88	67.1 ± 8.7 41 – 79	0.023
Follow-up (months)		mean ± SD range	21.6 ± 13.1 5 – 51	21.9 ± 13.8 5 – 52	0.903	21.8 ± 13.3 5 – 52	19.4 ± 10.1 6 – 41	0.380
Sex	m	n (%)	250 (65.8)	26 (65.0)	0.920	265 (66.9)	11 (45.8)	0.035
	f	n (%)	130 (34.2)	14 (35.0)		131 (33.1)	13 (54.2)	
vBMD	T5-8	mean ± SD	132.6 ± 40.7	110.6 ± 40.0	0.001	132.5 ± 40.5	97.4 ± 37.2	<0.001
(mg/cm^3^ CaHA)	T9-12	mean ± SD	126.8 ± 38.2	105.4 ± 34.1	0.001	126.5 ± 38.0	95.9 ± 31.5	<0.001
	L1-5	mean ± SD	121.1 ± 35.6	96.2 ± 29.9	<0.001	120.7 ± 35.3	87.5 ± 29.5	<0.001

*t-test for continuous variables (age, vBMD), chi2 for nominal variables (sex).

SD, Standard deviation; prev. VF, prevalent vertebral fracture; inc. VF, incident vertebral fracture; vBMD, volumetric bone mineral density; CaHA, calcium hydroxy-apatite.

**Figure 3:**
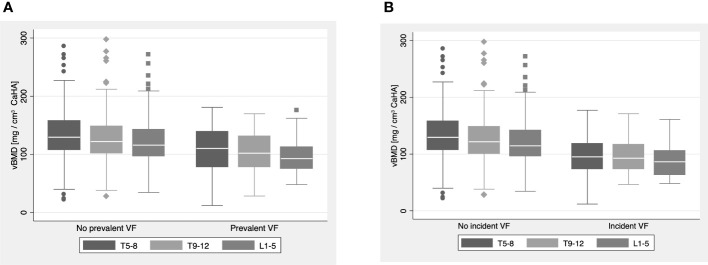
**(A, B)** Boxplots visualizing volumetric bone mineral density measurements (vBMD) at the T5-8, T9-12 and L1-5 level for the **(A)** ‘no prevalent VF’ and ‘prevalent VF’ cohorts and **(B)** ‘no incident VF’ and ‘incident VF’ cohorts. Throughout all levels, vBMDs in the ‘prevalent VF’ and ‘incident VF’ cohorts were significantly lower compared to their counterpart without VFs (p ≤ 0.001).

Prevalent VFs were detected in 40 out of 420 individuals (9.5%), and individuals with prevalent fractures had lower vBMD compared to those without (p ≤ 0.001) (**Figure 3A**). Of all individuals with prevalent VFs, 14 had multiple VFs for a total of 66 fractures. Twenty-four individuals (5.7%) suffered incident compression fractures during the follow-up interval, and the cohort with incident fractures had significantly lower vBMD than the cohort without (p < 0.001) (**Figure 3B**). Outliers in vBMD measurements (shown in **Figures 3A, B**) were on average 50.6 years old and did not show a clear sex predominance (60% male), when compared to the entire cohort. In non-fractured individuals, the highest rate of outliers was found at the T5-8 level (n = 8; 2%), while only one measurement outlier was found for patients with a prevalent fracture (L1-L5 vBMD). Manual review of outliers’ MDCT scans and charts did not reveal underlying bone diseases or artifacts.

### 3.2 Associations between baseline vBMD and prevalent VFs

Across sexes, VF prevalence was similar (male: 10.4%, female: 10.7%, p = 0.92 and 1.00), but individuals with VFs were older than those without (67.3 ± 9.8 years vs. 62.5 ± 9.2 years, p = 0.002) (**Table 1**). Thus, to account for confounding, multivariate analyses included age as a predictor.

**Figure 4:**
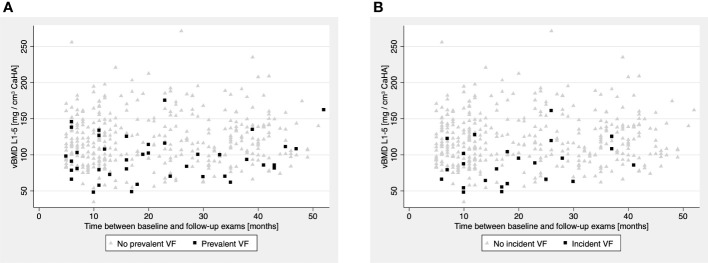
**(A, B)** Volumetric bone mineral density (vBMD) at the L1-5 level plotted against the time between baseline and follow-up scan in months. **(A)** Patients with prevalent VFs (black squares) were predominantly observed in the lower vBMD range, compared to patients without prevalent VFs (light-grey triangles). **(B)** Analogously, patients with incident VFs (black squares) were predominantly observed in the lower vBMD range, compared to patients without incident VFs (light-grey triangles).

Through all vertebral levels, lower vBMD was associated with higher odds for prevalent VFs and ORs increased gradually in a cranio-caudal direction (**Table 2**): vBMD at the lumbar spine (L1-5) was the strongest predictor of prevalent VFs (OR_SD_ = 2.21, 95%-CI_SD_ = 1.38 – 3.51, p = 0.001). A greater number of prevalent VFs was also strongly associated with lower vBMD across all vertebral levels: the strongest association was again found for the lumbar spine, but a cranio-caudal increase in coefficients was not observed (L1-5: Coef_SD_ = -0.103, 95%-CI_SD_ = -0.166 – 0.039, p = 0.002) (**Table 3** and **Figure 4A**).

**Table 2:** Prevalent VF prediction by vBMD.

	OR_SD_	95%-CI_SD_	OR	95%-CI	p
vBMD T5-8	1.60	1.10 – 2.32	1.01	1.00 – 1.02	0.015
vBMD T9-12	1.70	1.13 – 2.56	1.01	1.00 – 1.02	0.011
vBMD L1-5	2.21	1.38 – 3.51	1.02	1.01 – 1.04	0.001

All models are adjusted for age and sex.

vBMD, volumetric bone mineral density; VF, vertebral fracture OR, Odds ratio; 95%-CI, 95%-confidence interval.

_SD_ indicates values per standard deviation decrease in CaHA.

**Table 3:** Correlation of vBMD and prevalent VF count at baseline.

	Coef_SD_	95%-CI_SD_	Coef	95%-CI	p
vBMD T5-8	-0.083	-0.145 – -0.022	-0.002	-0.004 – -0.001	0.008
vBMD T9-12	-0.073	-0.134 – -0.012	-0.002	-0.004 – 0.000	0.020
vBMD L1-5	-0.103	-0.166 – -0.039	-0.003	-0.005 – -0.001	0.002

All models are adjusted for age and sex.

vBMD, volumetric bone mineral density; VF, vertebral fracture; Coef, Coefficient; 95%-C, 95%-confidence interval.

_SD_ indicates values per standard deviation decrease in CaHA.

### 3.3 Incident fracture prediction by baseline vBMD, VF status, and VF count

Females had a higher VF incidence rate than males (9.9% vs. 4.1%, p = 0.035 and 0.045, for chi-squared and Fisher’s exact test, respectively), and individuals suffering incident VFs were older compared to those, who did not have incident VFs (67.1 ± 8.7 years vs. 62.7 ± 9.3 years, p = 0.02) (**Table 1**). Thus, sex and age were both treated as confounders and included as predictors in all following multivariate models.

Although odds for incident VFs were greater in individuals with prevalent VFs and a higher VF count at baseline, multivariate models for both of these variables did not reach statistical significance (VF status: OR_SD_ = 2.15, 95%-CI = 0.72 – 6.43, p = 0.170; VF count: OR = 1.38, 95%-CI = 0.89 – 2.12, p = 0.147) (**Table 4**). Contrasting these findings, models based on opportunistic vBMD measurements showed that, through all considered vertebral levels, individuals with lower vBMD had significantly greater odds for incident VFs (T5-8, T9-12, L1-5: OR_SD_ range = 1.48 – 3.54, p ≤ 0.001, respectively), and all findings withstood the additional adjustment for baseline VF status (**Table 5**) (**Figure 4B**).

**Table 4:** Incident VF prediction by prevalent VF status and -count.

	OR	95%-CI	p	AIC	BIC
VF prevalence	2.15	0.72 – 6.43	0.170	181.0	197.1
VF count	1.38	0.89 – 2.12	0.147	180.7	196.9

All models are adjusted for age and sex.

VF, vertebral fracture; OR, Odds ratio; 95%-CI, 95%-confidence interval; AIC, Akaike’s information criterion; BIC, Bayesian information criterion.

**Table 5:** Incident VF prediction by vBMD.

	OR_SD_	95%-CI_SD_	p	OR	95%-CI	AIC	BIC
vBMD T5-8	2.45*	1.48 – 4.07	< 0.001	1.02	1.01 – 1.03	168.6	184.8
	2.38**	1.43 – 3.96	0.001	1.02	1.01 – 1.03	170.2	190.4
vBMD T9-12	2.58*	1.47 – 4.54	0.001	1.03	1.01 – 1.04	170.3	186.4
	2.50**	1.41 – 4.40	0.002	1.02	1.01 – 1.04	171.7	191.8
vBMD L1-5	3.54*	1.82 – 6.86	< 0.001	1.04	1.02 – 1.05	165.2	181.4
	3.42**	1.75 – 6.70	< 0.001	1.03	1.02 – 1.05	166.9	187.1

*Models are adjusted for age and sex.

**Models are adjusted for age, sex and baseline VF status.

vBMD, volumetric bone mineral density; VF, vertebral fracture; OR, Odds ratio; 95%-CI, 95%-confidence interval; AIC, Akaike’s information criterion; BIC, Bayesian information criterion.

_SD_ indicates values per standard deviation decrease in CaHA.

Model comparisons showed similar fit of models based on VF status and count (VF status: AIC = 181.0, BIC = 197.1; VF count: AIC = 180.7, BIC = 196.9). Fit of vBMD-based models was superior compared to models based on baseline VF status and VF count, and the model based on vBMD for L1-L5 fitted best (AIC = 165.2, BIC = 181.4).

## 4 Discussion

This study investigated whether opportunistic vBMD measurements obtained by an automated CNN-based framework predict future VFs and compared the developed prediction models with established models, which are based on VF prevalence. The major finding was that VF prediction using opportunistic vBMD-based models was found to be superior to prediction based on prevalent VFs. This may highlight the potential role of systematic, opportunistic vBMD assessments in addressing the current challenges of osteoporosis treatment and diagnosis.

While the overall prevalence rate of VFs was similar in females (10.7%) and males (10.4%), a strong imbalance was observed in VF incidence: females suffered 54.2% of VFs, although they represented only 34.3% of the cohort. These findings underline the well-known observation that females are at higher risk of osteoporosis and associated fractures, particularly during the post-menopause decades of life (8, 9, 33, 34).

Lumbar spine vBMD was most valuable for predicting incident VFs. Odds for incident VFs were increased 3.5-fold in individuals with a vBMD of -1 SD compared with the rest of the cohort. These results are comparable to previous studies that reported ORs of 2-6 or hazard ratios of 2.5-4.4 per SD vBMD decrease, respectively (14, 22, 35–37). However, previous studies focusing on vBMD measurements at the lumbar spine were limited by their smaller cohort sizes of 84-105 individuals (14, 22, 35). In contrast, Therkildsen et al. investigated 1487 patients but manually derived vBMD measurements from noncontrast-enhanced cardiac CTs covering only the thoracic spine and (36). While Johannesdottir et al. additionally analyzed lumbar vBMD at the L1-2 level, the authors’ approach with manual quantitative CT measurements limited to specific vertebrae was still inherently different from ours (37). The current approach combines the strengths of a larger cohort of 420 subjects, automated opportunistic vBMD extraction and vBMD extraction from the thoracic and lumbar spine.

A number of studies have previously investigated risk factors for imminent VFs and found that prevalent VFs are a strong predictor thereof (3–7). This finding is addressed in the FRAX^®^ tool (http://www.shef.ac.uk/FRAX), which queries fracture history in a dedicated question and includes it as an independent risk factor in the fracture risk calculation (11). Although individuals with prevalent VFs were more likely to suffer incident VFs than those without prevalent VFs with an OR of 2.15, the fracture-based models did however not reach statistical significance. This observation was confirmed by AIC and BIC: Both tests yielded lower scores for vBMD-based models, suggesting a superior fit (38). While in clinical practice, assessing fracture history through anamnesis is uncomplicated and provides valuable insights into bone stability and should therefore be performed whatsoever, vBMD-based fracture prediction models may particularly be useful in individuals with silent (i.e., clinically unrecognized) fractures (12).

We need to acknowledge some limitations of our present study. All scans were performed with a single scanner, which may lead to questions whether our findings are reproducible using other scanners. Previous studies that investigated the tool used for automatic vBMD extraction have addressed this question and showed that findings between scanners were indeed comparable, when appropriately calibrated (14, 22, 24). Another limitation of this study is the cohort composition. As mentioned above, more than half of the included individuals were males, despite the observation that females are more frequently affected by osteoporosis and VFs. This problem may have been avoided by frequency matching for sex (and age). However, this would have reduced our cohort size, which we intended to avoid. Further, frequency matching may induce new bias, as the cohorts may become too similar, which affects the observable effect size (39, 40). Instead, we decided to address this topic by including both variables (sex and age) as confounders in our multivariate analysis, where still statistically significant differences between the cohorts with and without VFs were found. While we did find vBMD outliers in our data (**Figures 3A, B**), we could not identify underlying confounders. On one side, vBMD measurements with asynchronous external calibration are known to be influenced by spine positioning in relation to the gantry due to x-ray field inhomogeneities and differences in beam hardening dependent on the scanner’s isocenter (41, 42). On the other side, chart data on bone active medications may be incomplete, introducing a possible bias to our findings. The inclusion criteria for this study may have resulted in a mainly oncologic patient population. Although we ruled out bone metastases during chart and imaging review, oncologic patients are nonetheless known to be at increased risk of bone loss and subsequent fractures, which limits the generalizability of our findings to non-oncologic populations. While our study highlights the potential role of opportunistic vBMD measurements in VF prediction, dual x-ray absorptiometry remains the gold standard for the diagnosis of osteoporosis and is implemented into current fracture prediction tools, like FRAX® (11, 19). However, MDCT scan numbers have drastically increased over the past 20 years (43). Therefore, opportunistic vBMD measurements gain increasing relevance, particularly for patients at risk of bone loss, like oncologic patients.

Prediction of VFs based on opportunistic vBMD assessments with an automated CNN-based framework is feasible and could particularly improve fracture prediction and detection in individuals receiving regular CT scans for oncologic staging. Moreover, vBMD was a stronger predictor of imminent VFs than fracture history. Opportunistic vBMD measurements from an automated framework for vertebral body segmentation and vBMD extraction could therefore enable fracture prevention measures.

## Data availability statement

The raw data supporting the conclusions of this article will be made available by the authors, without undue reservation.

## Ethics statement

The studies involving human participants were reviewed and approved by Ethikkommission der Technischen Universität München. Written informed consent for participation was not required for this study in accordance with the national legislation and the institutional requirements.

## Author contributions

JB: Methodology, Formal analysis, Investigation, Data Curation, Writing - Original Draft, Visualization. MD: Conceptualization, Software, Data Curation, Writing – Review & Editing. NS: Writing – Review & Editing. EB: Writing – Review & Editing. SR: Writing – Review & Editing. ML: Software, Data curation, Writing – Review & Editing. AS: Writing – Review & Editing. MH: Writing – Review & Editing. CZ: Writing – Review & Editing, Resources, Supervision. JK: Writing – Review & Editing, Funding acquisition. TB: Conceptualization, Resources, Data Curation, Writing – Review & Editing, Supervision, Project administration, Funding acquisition. All authors contributed to the article and approved the submitted version.
